# High Seroprevalence of Hepatitis E Virus Among Patients With Hepatitis B and Those With Hepatitis of Unknown Etiology in Central Vietnam

**DOI:** 10.1093/ofid/ofaf381

**Published:** 2025-06-26

**Authors:** Le Chi Cao, Tran Thi Tien Xinh, Taanvi Gowdar, Dang Ngoc Phuoc, Nguyen Thi Dung, Tran Thi Kim Loan, Pham Van Duc, Dao Thi Huyen, Le Thi Kieu Linh, Le Huu Song, Thirumalaisamy P Velavan

**Affiliations:** Institute of Tropical Medicine, University of Tübingen, Tübingen, Germany; Hue University of Medicine and Pharmacy (HUMP), Hue University, Hue, Vietnam; Hue University of Medicine and Pharmacy (HUMP), Hue University, Hue, Vietnam; Institute of Tropical Medicine, University of Tübingen, Tübingen, Germany; Hue University of Medicine and Pharmacy (HUMP), Hue University, Hue, Vietnam; Hue University of Medicine and Pharmacy (HUMP), Hue University, Hue, Vietnam; Hue University of Medicine and Pharmacy (HUMP), Hue University, Hue, Vietnam; Hue University of Medicine and Pharmacy (HUMP), Hue University, Hue, Vietnam; Vietnamese-German Center for Medical Research (VG-CARE), Hanoi, Vietnam; Institute of Tropical Medicine, University of Tübingen, Tübingen, Germany; Institute of Tropical Medicine, University of Tübingen, Tübingen, Germany; Vietnamese-German Center for Medical Research (VG-CARE), Hanoi, Vietnam; 108 Military Central Hospital, Hanoi, Vietnam; Institute of Tropical Medicine, University of Tübingen, Tübingen, Germany; Vietnamese-German Center for Medical Research (VG-CARE), Hanoi, Vietnam; Faculty of Medicine, Duy Tan University Danang, Vietnam

**Keywords:** hepatitis b, hepatitis e, hepatitis of unknown etiology, liver disease, seroprevalence

## Abstract

**Background:**

Hepatitis E virus (HEV) is a significant cause of acute viral hepatitis, particularly in regions with poor sanitation. Co-infection with hepatitis B virus (HBV) is common in high-risk populations, such as individuals with chronic liver disease, but data on HEV prevalence in patients living with HBV in Southeast Asia are limited. This study investigates HEV seroprevalence among patients with HBV and those with hepatitis of unknown etiology (HUE) in central Vietnam

**Method:**

Blood samples from 587 patients with HBV and 158 individuals with HUE were collected and analyzed for anti-HEV immunoglobulin G (IgG) and immunoglobulin M antibodies by enzyme-linked immunosorbent assay. HEV RNA was detected using reverse transcriptase-polymerase chain reaction, and HBV viral load was quantified via real-time polymerase chain reaction.

**Results:**

The overall anti-HEV IgG seroprevalence was 26% in patients with HBV and 36% in those with HUE. Although patients with liver cirrhosis and hepatocellular carcinoma exhibited higher rates of anti-HEV IgG seropositivity, these differences were not statistically significant. Among patients with HBV, the seroprevalence was highest in those with liver cirrhosis and hepatocellular carcinoma (46%), whereas the lowest (24%) was found in patients with symptomatic chronic hepatitis B. Only 1 (0.1%) tested positive for anti-HEV immunoglobulin M, and no HEV RNA positivity was detected. Furthermore, biochemical laboratory parameters in patients with liver diseases were not found to be associated with anti-HEV seropositivity.

**Conclusions:**

Anti-HEV IgG seroprevalence was relatively high among patients with chronic liver disease, including those with HBV. However, no association was observed between HEV exposure and liver disease progression. HEV is not a common cause of hepatitis in Central Vietnam.

Hepatitis E virus (HEV) is a major cause of acute viral hepatitis, particularly in regions with poor sanitation and hygiene conditions, which are common in low- and middle-income countries, and in rural areas where pigs, a major reservoir of HEV, are often reared. Every year, an estimated 20.1 million people are infected with the HEV, of which around 3.4 million become symptomatic, resulting in >70,000 deaths and 3000 stillbirths annually [1]. Although HEV often resolves without medical intervention, it poses a significant risk to certain high-risk groups, including pregnant women, organ transplant recipients, people with chronic liver disease, and those with HIV [2, 3]. In addition, HEV infections can lead to severe extrahepatic complications such as neurological manifestations and glomerulonephritis [4].

HEV is a small RNA virus with a 7.2-kb genome, comprising 3 distinct open reading frames. The virus exists in 2 forms: quasi-enveloped particles that circulate in the bloodstream and nonenveloped virions excreted in feces [5]. The virus belongs to the family *Hepeviridae* and the subfamily *Orthohepevirinae*, which includes 4 genera. The genus *Paslahepevirus*, which is associated with human and zoonotic HEV, contains the species *Paslahepevirus balayani* with 8 distinct genotypes (HEV-1 to HEV-8) [6]. Genotypes 1 and 2 are human-specific pathogens, predominantly transmitted via the fecal-oral route, and are most common among regions with inadequate and/or poor sanitation. In contrast, genotypes 3, 4, and 7 are zoonotic, infecting both humans and animals and are also prevalent in high-income countries [7]. Genotypes 5, 6, and 8 have been identified exclusively in animals to date [3].

In Southeast Asia, the burden of HEV has been investigated in several studies, with reported seroprevalence ranging from 2% among blood donors in Malaysia to 77.7% in lowland communities in Laos [8]. In particular, the co-infection and interactions between HEV, hepatitis B virus (HBV), and other chronic liver diseases have received increasing attention because of their potential to exacerbate liver pathology even in immunocompetent individuals [9, 10]. Given that SEA is also an endemic region for hepatitis B, with >61 million people chronically infected [11], continued research into HEV-HBV interactions remains essential. Vietnam, in particular, is recognized as a high endemicity region for HBV, with approximately 9% of the general population testing positive for hepatitis B surface antigen (HBsAg), making HBV a major contributor to liver cancer [12]. Additionally, prior exposure to HEV has been shown to influence the clinical course of chronic HBV infection, underscoring the important role of this HEV RNA virus in HBV treatment management and cryptogenic hepatitis [9, 13].

Vietnam is a well-recognized as a hotspot for zoonotic HEV infection [14, 15]. Epidemiological studies in the country have reported significant HEV exposure in the healthy population [9, 15], with prevalence ranging from 27% in the northern region to 31% in the southern region [16, 17]. In addition, Vietnam is among the leading countries in pig production and consumption [18], with pigs serving as a primary reservoir for HEV [15]. However, inadequate food safety measures and absence of effective control strategies may contribute to an increased risk of widespread HEV transmission [15]. Given these factors, there is a critical need for further serological surveillance of HEV, particularly among high-risk groups such as individuals with liver diseases, including those with HBV.

This study investigates the seroprevalence of HEV infection among individuals with HBV and those with hepatitis of unknown etiology (HUE) in Central Vietnam. The aim is to elucidate the burden of HEV in high-risk populations within this region and assess its potential impact on the clinical progression of HBV infection.

## MATERIALS AND METHODS

### Ethics Statement

The study was approved by the ethics committee of Hue University of Medicine and Pharmacy, Hue University, Vietnam (H2024/585). All experiments were performed following Good Clinical Laboratory Practice guidelines.

### Study Design and Collection

Blood samples were collected from both inpatients and outpatients at the Hospital of Hue University of Medicine and Pharmacy, Central Vietnam, between June 2023 and June 2024. The blood samples were initially obtained as part of routine diagnostic procedures during standard medical care. With approval from the institutional ethics committee, a portion of each sample was later used for research purposes in this study. All patients provided written informed consent before participation. For patients who tested positive for HEV-RNA or HEV-immunoglobulin M (IgM), the results were forwarded to the responsible physicians to ensure appropriate clinical treatment. A total of 587 samples were obtained from individuals with HBV, confirmed by a positive HBsAg result, including those undergoing treatment for HBV. In addition, 158 blood samples were collected from patients with HUE (non-B and non-C hepatitis). Clinical manifestations and laboratory parameters were obtained from the medical records of all enrolled patients for comprehensive analysis.

HBV infection was classified according to World Health Organization guidelines. Acute hepatitis B (n = 3) was defined as HBsAg positivity for <6 months, with anti-HBc IgM positivity, elevated liver enzymes (typically more than 5 times the upper limit of normal), with no history of previous HBV infection. Chronic hepatitis B (CHB, n = 584) was characterized by HBsAg positivity for more than 6 months and anti-HBc immunoglobulin G (IgG) positivity [19]. To assess how HEV exposure influences the progression of HBV infection, patients with CHB were stratified into the following clinical categories: (1) symptomatic CHB (SYMP + CHB) included individuals with current or prior hepatitis symptoms and elevated liver enzyme levels, but without evidence of liver cirrhosis (LC) or hepatocellular carcinoma (HCC); (2) CHB with liver cirrhosis (LC + CHB) and (3) CHB with hepatocellular carcinoma (HCC + CHB) were defined by the presence of LC or HCC, respectively; (4) CHB with both LC and HCC (LC + HCC + CHB) included patients diagnosed with both complications; (5) asymptomatic CHB (ASYMP + CHB) comprised individuals with HBV infection lasting more than 6 months, but without clinical symptoms or elevated liver enzymes. Similarly, 158 patients with HUE were categorized into 4 subgroups: (1) symptomatic hepatitis without liver cirrhosis or hepatocellular carcinoma (SYMP + HUE); (2) HUE with liver cirrhosis (LC + HUE); (3) HUE with hepatocellular carcinoma (HCC + HUE); and (4) HUE with both liver cirrhosis and hepatocellular carcinoma (LC + HCC + HUE).

Cirrhotic patients were identified based on clinical symptoms, and biochemical abnormalities including hyperbilirubinemia, elevated aspartate aminotransferase (AST) and alanine aminotransferase (ALT) levels, prolonged prothrombin time, decreased serum albumin levels, and ultrasonographic evidence of chronic liver parenchymal damage. In addition, the diagnosis of HCC was based on clinical manifestations, imaging findings, and histopathological confirmation.

### HEV Serological Assays

Serum samples were analyzed for anti-HEV IgG and anti-HEV IgM antibodies using enzyme-linked immunosorbent assay kits (WANTAI, Beijing, China). The anti-HEV IgG assay (sensitivity: 99.08%; specificity: 99.9%) and the anti-HEV IgM assay (sensitivity: 97.1%; specificity: 98.4%) were previously validated in multicenter clinical studies as provided by the manufacturer (https://www.dbaitalia.it/newsletter/we–7296_hev). Testing was performed according to the manufacturer's protocol. Absorbance was measured using a CLARIOstar microplate reader (BMG Labtech, Rotenberg, Germany).

### Nucleic Acid Isolation

Nucleic acid isolation from serum samples was performed using the QIAamp Viral RNA Mini Kit (Qiagen GmbH, Hilden, Germany) for HEV RNA extraction and the QIAamp Viral DNA Mini Kit (Qiagen GmbH) for HBV DNA extraction according to the manufacturer's instructions. A total of 140 µL of serum was used for each isolation and the nucleic acids were eluted in 80 µL of elution buffer. The extracted products were stored at −80°C until used.

### Reverse Transcriptase-Polymerase Chain Reaction for HEV-RNA Detection

HEV RNA was reverse transcribed into cDNA using the High-Capacity cDNA Reverse Transcription Kit (ThermoFisher Scientific, Waltham, MA, USA). The presence of HEV RNA was detected using a nested polymerase chain reaction (PCR) assay targeting the conserved regions of HEV ORF1, as previously described by Hoan et al [15]. The outer primers were HEV-38 (5′-GAGGCYATGGTSGAGAARG-3′) and HEV-39 (5′-GCCATGTTCCAGACRGTRTTCC-3′), while the inner primers were HEV-37 (5′-GGTTCCGYGCTATTGARAARG-3′) and HEV-27 (5′-TCRCCAGAGTGYTTCTTCC-3′). PCR amplification was performed in a 25-µL reaction containing 5 ng cDNA, PCR buffer, dNTPs, MgCl2, specific primers as indicated previously (Eurofins Genomics, Ebersberg, Germany), and Taq polymerase (Qiagen GmbH). Thermal cycling parameters for the outer and nested PCRs included initial denaturation at 94°C for 5 minutes, followed by 35 cycles of denaturation (94°C for 30 seconds), annealing (54°C for PCR outer or 56°C for PCR inner in 30 seconds), and extension (72°C for 30 seconds), with a final extension for 5 minutes at 72°C. A plasmid containing HEV cDNA was used as a positive control. Amplicons (306 bp) were visualized on 1.2% agarose gels stained with SYBR Green.

### Quantitative Detection of HBV

Real-time PCR targeting a conserved 90-bp region of the HBV S gene (GenBank #X75657, position 182–271) was performed using the SensiFAST One-Step RT-PCR Kit (Meridian Biosciences, Memphis, TN, USA) on a LightCycler 480-II (Roche, Mannheim, Germany). Each 20-μL reaction contained 10 μL of 2× master mix, 0.8 μL of each 10 μM primer (Eurofins Genomics, Ebersberg, Germany) (forward: HBV-61 (5′-GGACCCCTGCTCGTGTTACA-3′); reverse: HBV-62 (5′-GAGAGAAGTCCACC ACGAGTCTAGA-3′), 0.3 μL of 10 μM probe HBV-TM-05 (FAM-5′-TGTTGACAARAATC CTCACAATACCRCAGA-3′-DabCyl) and 5 μL (15–20 ng) of DNA. Cycling conditions were 95°C for 5 minutes followed by 45 cycles of 95°C for 10 seconds and 60°C for 34 seconds. The detection limit of the assay was 25 IU/mL, calibrated with a 10⁶ copies/μL plasmid standard and 10-fold dilutions. Cycle threshold values were used to calculate viral load, with a maximum cycle threshold value of 40 corresponding to 221 copies/mL.

### Statistical Analysis

All analyses were performed using GraphPad Prism (version 9.5.1), with a *P* value <.05 considered statistically significant. Quantitative data were presented as median with ranges, and categorical data as counts and percentages. Categorical variables were analyzed using chi-squared or Fisher exact tests, whereas continuous variables were analyzed using *t*-tests or Kruskal-Wallis tests. Multivariate analyses adjusted for age and sex were performed using SPSS (version 20).

## RESULTS

### Demographic Data and Clinical Characteristics of Study Cohorts

The demographic characteristics of 587 patients with HBV and 158 patients with HUE are presented in Table 1. The median age of patients with HBV and patients with HUE were 50 (range, 5–97 years) and 55.5 (10–83 years) respectively. The male/female ratio of patients with HBV and patients with HUE were 331/256, and 107/51, respectively. Among the 587 patients with HBV, the majority were diagnosed with CHB (n = 584), whereas a small number had acute hepatitis B (n = 3). Of the 584 patients with CHB, most were symptomatic (SYMP + CHB; n = 426), followed by asymptomatic individuals (ASYMP + CHB; n = 88). Additional subgroups within the CHB cohort included those with liver cirrhosis (LC + CHB; n = 43), hepatocellular carcinoma (HCC + CHB; n = 14), and both liver cirrhosis and hepatocellular carcinoma (LC + HCC + CHB; n = 13) (Table 1). Among 158 patients with HUE, the majority had liver cirrhosis (LC + HUE; n = 79), followed by symptomatic hepatitis (SYMP + HUE; n = 58), hepatocellular carcinoma (HCC + HUE; n = 11), and both LC and HCC (LC + HCC + HUE; n = 10) (Table 2). Significant differences were observed in the HBV subgroups with regard to age, red blood cells, platelets, AST, ALT, prothrombin time, and alpha-fetoprotein (AFP). In the HUE subgroups, age, platelets, prothrombin time, and AFP differed significantly, whereas liver enzymes showed no statistical differences.

**Table 1.** Demographic and Clinical Characteristics of Patients Living With Hepatitis B Infection

	Patients With Hepatitis B (n = 587)					
	SYMP + CHB (n = 426)	ASYMP + CHB (n = 88)	LC + CHB (n = 43)	HCC + CHB (n = 14)	LC + HCC + CHB (n = 13)	AHB (n = 3)
Age (y)*	50 [5–97]	44 [14–89]	56 [26–79]	70 [40–77]	58 [29–68]	34 [21–43]
Sex ratio (M/F)	232/194	42/46	30/13	12/2	12/1	3/0
WBC (10^3^/µL)	6.6 [2.4–20.0]	7.0 [4.4–19.5]	6.3 [2.7–12.4]	6.8 [2.9–11.1]	7.5 [4.6–19.4]	7.8 [5.7–8.6]
RBC (10^6^/µL)*	4.6 [2.8–7.2]	4.6 [2.9–6.9]	4.2 [2.6–5.3]	4.4 [3.0–5.5]	4.2 [3.3–4.6]	4.3 [3.9–4.5]
Hb (g/L)	140 [82–179]	139 [63–184]	130 [91–175]	144 [94–170]	129 [89–153]	133 [117–152]
PLT (10^3^/µl)*	201 [43–597]	239 [129–546]	148 [39–415]	192 [91–508]	152 [103–278]	129 [64–188]
AST (IU/L)*	31 [14–737]	23 [12–53]	47 [18–392]	40 [24–72]	80 [29–660]	405 [344–1504]
ALT (IU/L)*	30 [6–513]	23 [7–56]	34 [14–517]	38 [10–88]	87 [21–741]	949 [716–2053]
Prothrombin time (s)*	12.6 (3.1–38.4)	12.2 [9.3–16.9]	12.9 [9.7–25.4]	13.7 [11.1–17.1]	14.0 [12.4–25.9]	15.9 [10.9–18.6]
AFP (ng/mL)*	3 [1–195]	2 [1–8]	10 [1–280]	17.2 [3–29136]	864 [3–14727]	n/a
HBV viral loads (copies/mL)	1431 [221–3.2 × 10^9^]	2802 [221–3.1 × 10^8^]	1142 [221–4.1 × 10^8^]	4.7 × 10^4^ [284–1.2 × 10^7^]	1112 [221–6.2 × 10^6^]	1.5 × 10^6^ [4.1 × 10^5^–2.8 × 10^6^]

Values are presented as medians and ranges. HBV quantification limit: 221 copies/mL (Ct 40).

Abbreviations: AFP, alpha-feto protein; AHB, acute hepatitis B; AST and ALT, aspartate and alanine amino transferase; ASYMP, Asymptomatic; CHB, chronic hepatitis B; F, female; HCC, hepatocellular carcinoma; LC, liver cirrhosis; M, male; n/a, not applicable; PLT, platelets; RBC, red blood cells; SYMP, symptomatic; WBC, white blood cells.

^*^ *P* < .001 for comparisons with all other groups using a 1-way analysis of variance test.

**Table 2.** Demographic and Clinical Characteristics of Patients With Hepatitis of Unknown Etiology (HUE)

	Hepatitis of Unknown Etiology (n = 158)			
	LC (n = 79)	SYMP (n = 58)	HCC (n = 11)	LC + HCC (n = 10)
Age (y)*	55 [33–83]	54 [10–81]	67 [55–80]	69 [37–82]
Sex ratio (M/F)	65/14	27/31	8/3	7/3
WBC (10^3^/µL)	5.9 [1.7–16.1]	7.3 [2.8–17.0]	6.7 [3.7–9.2]	6.9 [4.6–16.6]
RBC (10^6^/µL)*	4.0 [1.8–5.6]	4.4 [2.7–6.0]	4.6 [3.2–5.1]	4.4 [4.1–4.7]
Hb (g/L)	122 [47–195]	130 [90–160]	138 [98–161]	138 [121–150]
PLT (10^3^/µL)*	128 [11–332]	234 [76–437]	252 [131–461]	136 [79–375]
AST (IU/L)	64 [11–940]	40 [14–1550]	33 [14–82]	76 [23345]
ALT (IU/L)	37 [9–1543]	38 [3–1579]	26 [6–54.3]	51 [17–584]
Prothrombin time (s)*	13.6 [10.3–41.2]	12.2 [10.1–21.2]	11.4 [9.8–17.3]	13.5 [10.7–17.4]
AFP (ng/mL)*	4.7 [2–325]	3 [1–97]	5 [2–2697]	103 [2–8849]

Values are presented as medians and ranges.

Abbreviations: AFP, alpha-feto protein; AST and ALT, aspartate and alanine amino transferase; F, female; HCC, hepatocellular carcinoma; LC, liver cirrhosis; M, male; PLT, platelets; RBC, red blood cells; SYMP, symptomatic; WBC, white blood cells.

^*^ *P* < .001 for comparisons means of the independent groups using a 1-way analysis of variance test.

### Anti-HEV IgG Seroprevalence and RNA Positivity in HBV and HUE

**Figure 1.**
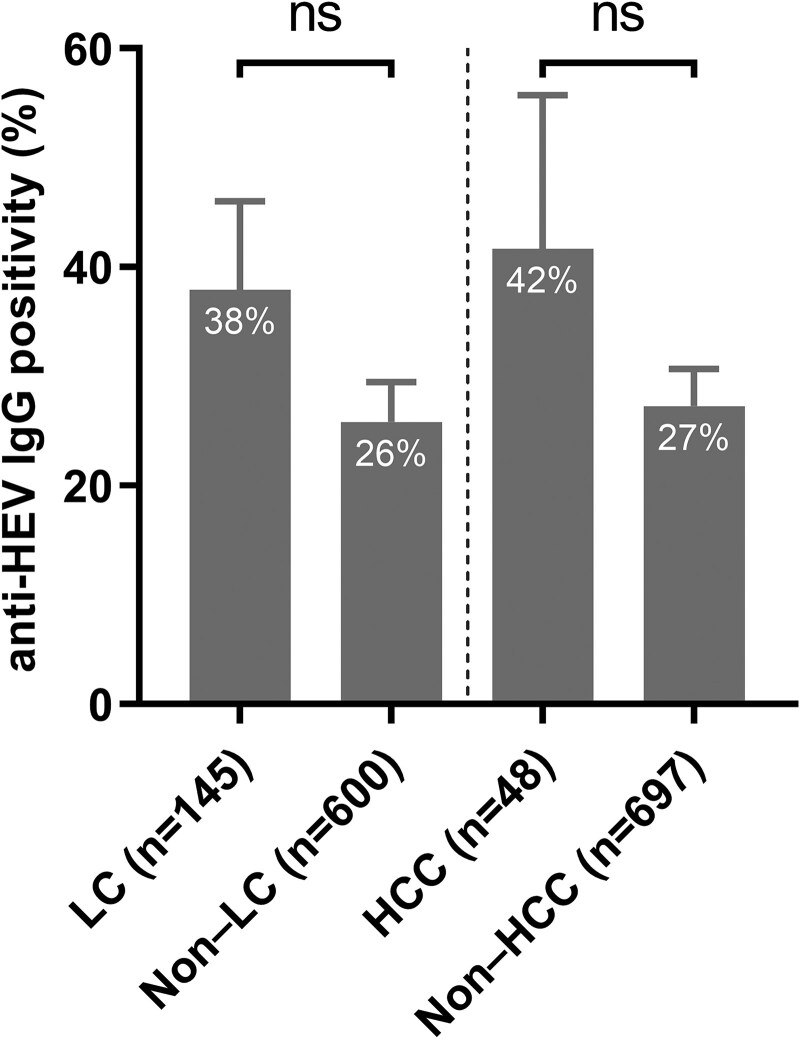
Anti-HEV IgG seroprevalence in patients with liver cirrhosis (LC) and hepatocellular carcinoma (HCC). *P* values were calculated using the chi-square test, adjusted for age and sex.

In this study population, anti-HEV IgG seroprevalence was high among both patients with HBV (26%, n = 153/587) and those with HUE (36%, n = 57/158) (Table 3). Among patients with HBV, anti-HEV IgG seropositivity was highest in those with both liver cirrhosis and hepatocellular carcinoma (46%, LC + HCC + CHB; n = 6/13) and those with hepatocellular carcinoma alone (43%, HCC + CHB; n = 6/14), followed by patients with liver cirrhosis (33%, LC + CHB; n = 14/43), asymptomatic CHB (28%, ASYMP + CHB; n = 25/88), and symptomatic CHB (24%, SYMP + CHB; n = 102/426) (Table 3). Among patients with hepatitis of unknown etiology, anti-HEV IgG seropositivity was highest in those with both liver cirrhosis and hepatocellular carcinoma (40%, LC + HCC + HUE; n = 4/10), followed by those with liver cirrhosis alone (39%, LC + HUE; n = 31/79), hepatocellular carcinoma alone (36%, HCC + HUE; n = 4/11), and symptomatic HUE (31%, SYMP + HUE; n = 18/58) (Table 3). No significant differences in anti-HEV IgG seropositivity were observed between the patients with HBV and HUE subgroups (*P* = .271), as age and sex were significantly associated with anti-HEV IgG seropositivity (*P* < .001) (Table 3). Seropositivity, which appeared to be higher in patients with liver cancer than in patients without liver cancer (38% vs 26%) and higher in patients with hepatocellular carcinoma than in patients without hepatocellular carcinoma (42% vs 27%), was also not significant after adjustment for age and gender (*P* > .05) (Figure 1). None of the patients with HUE tested positive for anti-HEV IgM, whereas 1 patient with LC + HBV was anti-HEV IgM and IgG positive. All 3 acute hepatitis B patients were negative for both anti-HEV IgG and IgM. No HEV viremia or HEV RNA was detected in the studied population.

**Table 3.** Anti-HEV IgG Seropositivity in Patients With HBV and Hepatitis of Unknown Etiology (HUE) Stratifying to Clinical Stages, Age, and Sex

	CHB % (n = 584)	HUE % (n = 158)	*P* Value
**Clinical stages**			*P* = .271^a^
LC + HCC	46% (6/13)	40% (4/10)	
HCC	43% (6/14)	36% (4/11)	
LC	33% (14/43)	39% (31/79)	
SYMP	24% (102/426)	31% (18/58)	
ASYM	28% (25/88)	n/a	
Total	26% (153/584)	36% (57/158)	
**Age group**			*P* < .001
<25	3% (1/32)	13% (1/8)	
25–34	11% (9/79)	14% (1/7)	
35–44	30% (32/108)	29% (5/17)	
45–54	24% (31/129)	48% (20/42)	
55–64	37% (38/102)	49% (20/41)	
65–74	35% (34/96)	24% (8/33)	
≥75	35% (8/38)	20% (2/10)	
**Gender**			*P* < .001
Male	31% (103/328)	44% (47/107)	
Female	20% (50/256)	20% (10/51)	

The *P* value for comparing IgG seropositivity among groups was calculated using logistic regression.

Abbreviations: ASYMP, asymptomatic; CHB, chronic hepatitis B; HCC, hepatocellular carcinoma; LC, liver cirrhosis; SYMP, symptomatic.

^a^ *P* value after adjusting age and sex.

### Association of HEV Infection With Biochemical Parameters in HBV and HUE Patients

**Figure 2.**
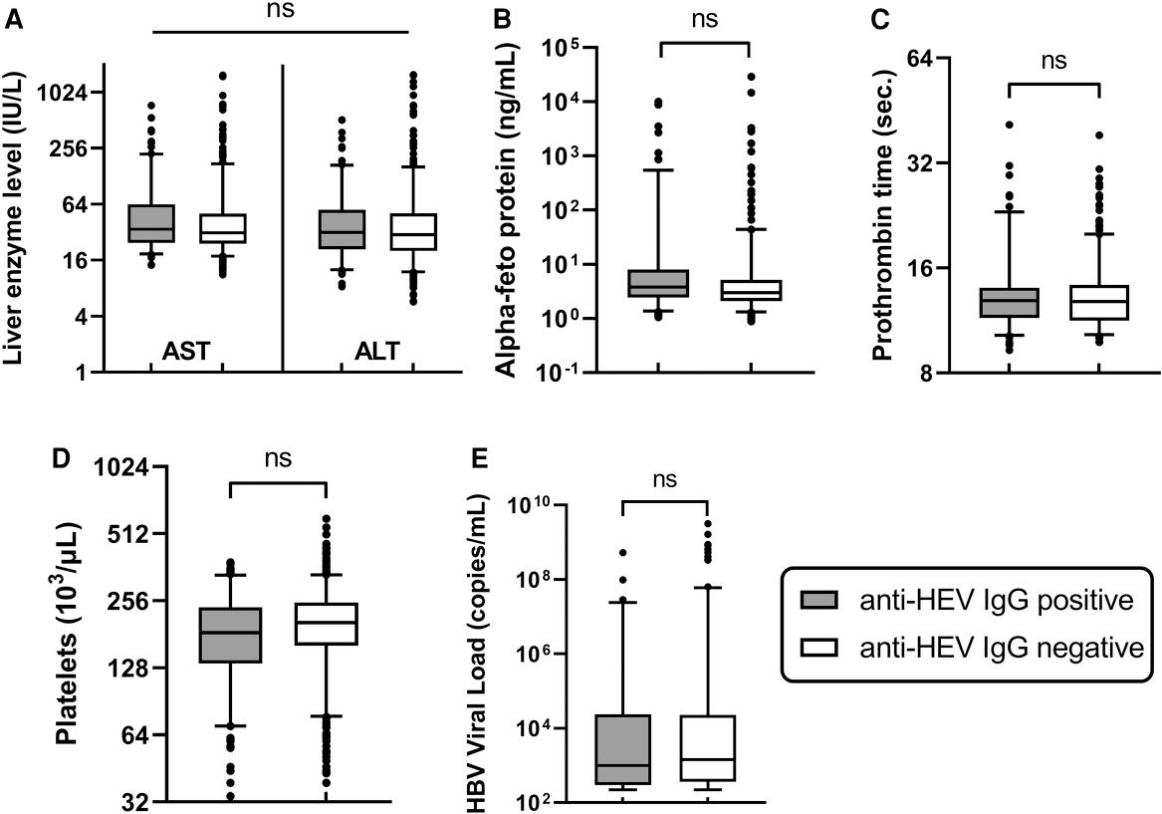
Comparison of biochemical parameters in anti-HEV IgG-positive and -negative patients. (*A*) Liver enzyme levels; (*B*) alpha-fetoprotein; (*C*) prothrombin time; (*D*) platelet count; (*E*) HBV viral load. ns, not significant. Box plots display medians with 25th and 75th percentiles and *P* values were calculated using multivariable logistic regression, adjusted for age and sex. HBV, hepatitis B virus; HEV, hepatitis E virus; IgG, immunoglobulin G.

**Figure 3.**
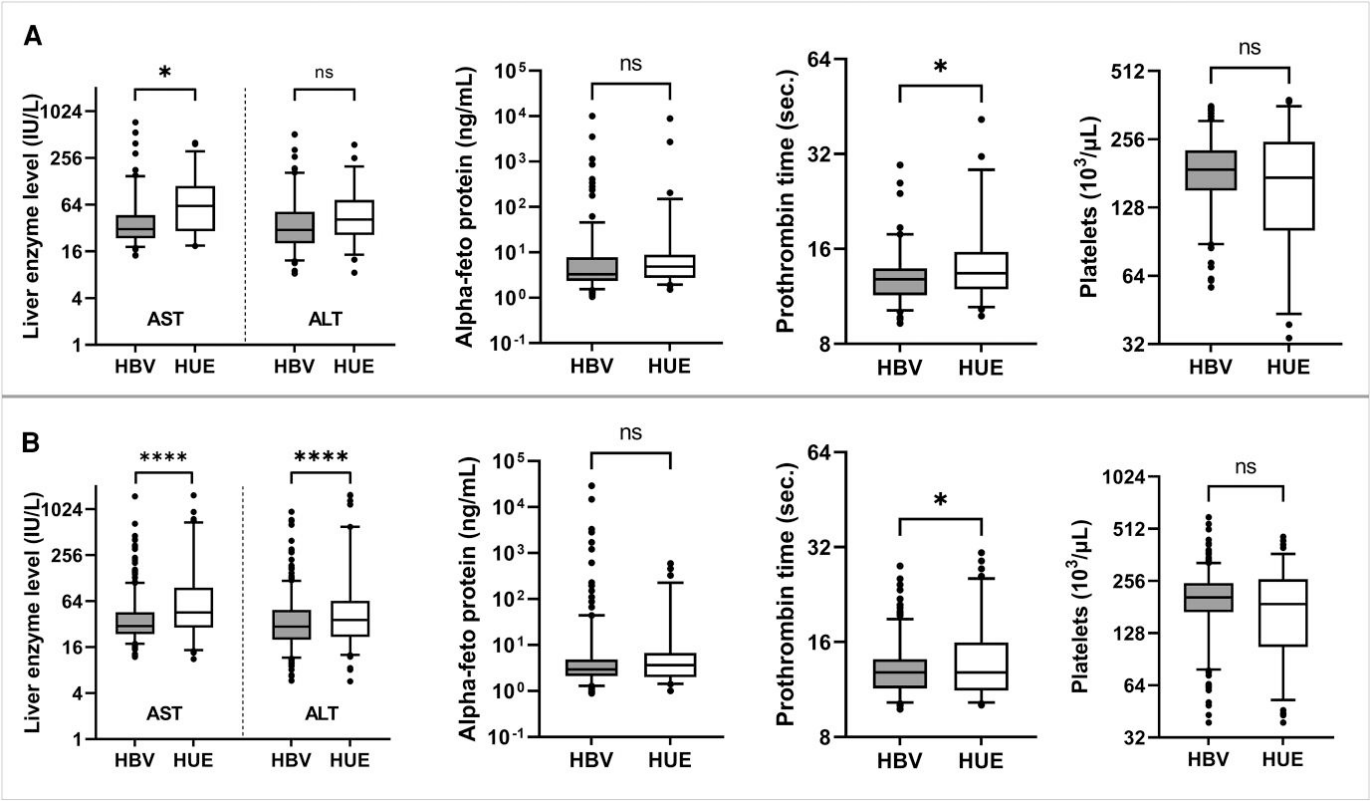
Biochemical parameters in patients with hepatitis B virus (HBV) and patients with hepatitis of unknown etiology (HUE). (*A*) Individuals positive for anti-HEV IgG positive; (*B*) individuals negative for anti-HEV IgG. ****P < .001. HEV, hepatitis E virus; IgG, immunoglobulin G.

The relationship between anti-HEV IgG seropositivity and various biochemical parameters including AST, ALT, AFP, prothrombin time, platelet count, and HBV viral load was examined. Although minor differences in these clinical laboratory values were observed between individuals with and without anti-HEV IgG, none was statistically significant after adjusting for age and sex (*P* > .05), as shown in Figure 2. Significant differences were observed in AST levels and prothrombin time (*P* < .05) between patients with HBV and HUE on anti-HEV IgG positivity, whereas ALT, AFP, and platelet count showed no significant differences (Figure 3*A*). Significant differences were observed in AST, ALT levels, and prothrombin time (*P* < .001) between patients with HBV and HUE who were HEV seronegative, whereas AFP and platelet count showed no significant differences (Figure 3*B*).

## DISCUSSION

HEV seroprevalence differs worldwide, reflecting diverse epidemiological patterns. A meta-analysis reported a 21% pooled anti-HEV IgG seroprevalence across Southeast Asia, with significant variation between countries. Myanmar had the highest (33.5%), followed by Vietnam (31.4%), whereas Malaysia had the lowest (5.9%) [20]. These differences are likely influenced by factors such as the pork consumption habits and the prevalence of pig farming in the region. Notably, both Vietnam and Myanmar are among the countries with high pork consumption, which is a key contributor to increased HEV exposure. In our study, IgG seropositivity ranged from 26% in patients with HBV to 36% in those with hepatitis of unknown etiology, consistent with previous reports from Northern and Southern Vietnam (27% among blood donors and 31.7% in hospital populations, respectively) [16, 17]. Northern Vietnam studies showed even higher seroprevalences (53% in occupationally exposed, 45% liver disease patients) [9, 15], suggesting Vietnam remains highly endemic for HEV with significant regional variations. Although previous studies have reported higher HEV seropositivity among patients with chronic liver diseases compared to the general population [20, 21], our findings indicate no significant difference in seropositivity between patients with HBV, HUE, and the general population in Vietnam.

In our study, the low prevalence of anti-HEV IgM, with only 1 case detected among 745 patients, indicates a low occurrence. A similar trend was observed in Vietnam, where IgM seropositivity was only 0.5%, despite a high 42% IgG prevalence [22]. Other studies in Vietnam have reported higher IgM rates, ranging from 2% in pregnant women [23], 3% in acute hepatitis [24], to 11%–12% in individuals occupationally exposed to pigs and with liver disease [9, 15]. In Southeast Asia, Cambodia and Laos had low IgM prevalence (around 1%), whereas Thailand and Indonesia reported higher rates (7.3%–12.4%), with Vietnam at 4.72% [20]. The difference in seropositivity between IgG and IgM in our study suggests a high prevalence of previous HEV exposure with limited evidence of ongoing transmission. The case of a patient with CHB who tested positive for both IgM and IgG but had undetectable HEV RNA suggests that the patient is in the later stages of acute or resolving hepatitis E infection, with the absence of HEV RNA, indicating a lack of active viral replication. Furthermore, the absence of IgM positivity in the HUE group supports the notion that HEV is a rare cause of hepatitis in Central Vietnam.

The seroprevalence of HEV in patients with HBV has been widely documented. A study in the United States and Canada found an anti-HEV IgG seroprevalence of 28.5% among 600 adults with CHB, similar to our findings, with no HEV RNA positivity [25]. In Vietnam, a study on pregnant women with HBV reported 26% IgG seroprevalence, suggesting comparable exposure across high-risk populations [26]. Another study in Vietnam reported higher rates (45% IgG, 12% IgM) in people living with HBV, with even higher rates in those with cirrhosis (54% IgG, 19% IgM) [9]. These variations may be due to differences in assay sensitivity, HBV disease progression, and geographical distribution of HEV infection [27]. A Chinese study reported lower seroprevalence, (9.1% IgG, 0.1% IgM) in patients with CHB, reflecting geographical differences in HEV exposure [28]. HEV superinfection in patients with HBV has been associated with increased mortality, particularly in cirrhotic patients [29]. Although anti-HEV IgG seropositivity was highest (46%) in patients with both LC and HCC, no significant difference was observed between those with and without these conditions. The elevated anti-HEV IgG seroprevalence in the LC/HCC group may be attributed to the older age and male predominance in this group, as both older individuals and males are more likely to have been exposed to HEV.

The relationship between anti-HEV IgG seropositivity and platelet count in patients with HBV is clinically significant, as platelet count is a known prognostic marker for HEV-related acute liver failure [30]. A study by Hoan et al found lower platelet levels in patients with HBV with HEV seropositivity compared to those without HEV exposure [9]. Although we observed lower platelet counts in anti-HEV IgG-positive patients compared to IgG-negative patients, this difference was not statistically significant after adjustment for age and gender. These results suggest that platelet counts are more strongly influenced by age and gender than by HEV exposure itself. We also analyzed other biochemical parameters such as AST and ALT, AFP, and prothrombin time. No significant differences were found between patients with anti-HEV IgG-positive and those without this antibody, which is in contrast to the results of studies in Vietnam and China [9, 31]. These discrepancies may be attributed to the high prevalence of current HEV infection in those studies and the high inclusion of patients with liver cirrhosis and hepatocellular carcinoma. We initially hypothesized that HEV exposure would influence biochemical parameters in patients with HBV. However, the comparable results observed between anti-HEV IgG-positive and -negative groups suggest that HEV exposure does not significantly impact the clinical parameters in patients with HBV. This finding indicates that, unlike other hepatitis co-infections, such as hepatitis D [32, 33], HEV may not exacerbate liver function abnormalities or disease progression in patients with HBV, underscoring the need for further studies to explore the interaction between these viruses in distinctive clinical contexts.

This study showed a high seroprevalence of anti-HEV IgG in patients with advanced liver disease, though it has several limitations. The small number of acute hepatitis B cases and the unequal size of the subgroups limit the evaluation of the role of HEV in different disease stages. In addition, the absence of anti-HEV IgG avidity results and lack of liver fibrosis data constrain the analysis of HEV exposure in relation to disease progression. In conclusion, although HEV exposure is common in patients with CHB and HUE with cirrhosis and/or HCC, disease progression appears to be more strongly related to age and gender than to HEV exposure. Furthermore, HEV does not appear to be a common cause of hepatitis in central Vietnam.

## Notes

***Acknowledgments.*** The authors thank all study participants for their participation. The authors also thank the staffs of the Hospital of Hue University of Medicine and Pharmacy for their support during sampling. They acknowledge support from the Open Access Publication Fund of the University of Tübingen.

***Author Contributions.*** T.P.V. and L.H.S. designed and supervised the study and contributed to the study materials and assays. L.C.C. and T.T.T.X. participated in the study design. L.C.C., T.T.T.X., D.N.P., N.T.D. and T.T.K.L. collected samples. L.C.C., T.G., P.V.D., D.T.H., and L.T.L.K. performed the experimental procedures. L.C.C. performed the statistical analysis and data visualization. L.C.C. wrote the first draft. T.P.V. and L.C.C. revised the first draft. All authors have read and approved the manuscript.

***Disclaimer.*** The funder has no role in the study design, data collection and analysis, decision to publish, or preparation of the manuscript.

***Data Availability.*** Not applicable.

***Financial support.*** This study was funded by the PAN-ASEAN Coalition for Epidemic and Outbreak Preparedness (PACE-UP; German Academic Exchange Service (DAAD) Project ID: 57592343).
